# Autoimmune diseases refractory to corticosteroids and immunosuppressants

**DOI:** 10.3389/fimmu.2024.1447337

**Published:** 2024-09-16

**Authors:** Zeev Elkoshi

**Affiliations:** Research and Development Department, Taro Pharmaceutical Industries Ltd, Haifa, Israel

**Keywords:** autoimmunity, immunosuppressants, type 1 diabetes, Hashimoto’s thyroiditis, Graves’ disease, primary biliary cholangitis

## Abstract

Corticosteroids and immunosuppressive drugs can alleviate the symptoms of most autoimmune diseases and induce remission by restraining the autoimmune attack and limiting the damage to the target tissues. However, four autoimmune non-degenerative diseases—adult advanced type 1 diabetes mellitus, Hashimoto’s thyroiditis, Graves’ disease, and advanced primary biliary cholangitis—are refractory to these drugs. This article suggests that the refractoriness of certain autoimmune diseases is due to near-total loss of secreting cells coupled with the extremely low regenerative capacity of the affected tissues. The near-complete destruction of cells responsible for secreting insulin, thyroid hormones, or biliary HCO_3_^−^ diminishes the protective effects of immunosuppressants against further damage. The slow regeneration rate of these cells hinders tissue recovery, even after drug-induced immune suppression, thus preventing remission. Although the liver can fully regenerate after injury, severe primary biliary cholangitis may impair this ability, preventing liver recovery. Consequently, these four autoimmune diseases are resistant to immunosuppressive drugs and corticosteroids. In contrast, early stages of type 1 diabetes and early primary biliary cholangitis, where damage to secreting cells is partial, may benefit from immunosuppressant treatment. In contrast to these four diseases, chronic degenerative autoimmune conditions like multiple sclerosis may respond positively to corticosteroid use despite the limited regenerative potential of the affected tissue (the central nervous system). The opposite is true for acute autoimmune conditions like Guillain–Barré syndrome.

## Introduction

Corticosteroids (CSs) are anti-inflammatory drugs that exert their effect by diffusing across membranes of cells that are involved with the inflammatory immune response, such as macrophages, eosinophils, lymphocytes, mast cells, and dendritic cells. Following membrane diffusion, corticosteroids bind to glucocorticoid receptors (GRs) in the cytosol of the cells, resulting in a conformational change in the receptor. The receptor–glucocorticoid complex then moves into the cell nucleus, where it dimerizes and binds to glucocorticoid response elements. The activated GR complex upregulates the expression of anti-inflammatory proteins in the nucleus or represses the expression of pro-inflammatory proteins in the cytosol. Corticosteroids inhibit genes responsible for the expression of cyclooxygenase-2, inducible nitric oxide synthase, and pro-inflammatory cytokines, including tumor necrosis factor-alpha and various interleukins. Another important effect is the inhibition of phospholipase A2, which is responsible for the production of numerous inflammatory mediators (1).

Corticosteroids are one member of a broader group of agents termed immunosuppressive drugs or immunosuppressants (ISs). Immunosuppressants are drugs that downregulate the innate and adaptive arms of the immune system. Small molecules that act as ISs include antimetabolic agents that interfere with DNA synthesis and cell proliferation (i.e., azathioprine, mycophenolic acid, and methotrexate), calcineurin inhibitors that block the transcription of nuclear factor of activated T cells (NFAT) with the result that T cells are maintained in the resting state of the cell cycle (i.e., cyclosporin A and tacrolimus), and mTOR inhibitors, which regulate cellular metabolism, growth, and proliferation by forming and signaling through two protein complexes, mTORC1 and mTORC2 (i.e., sirolimus). There are also polyclonal biologic preparations with immunomodulatory effects, such as intravenous immunoglobulin (IVIG). IVIG is a compound of plasma-derived immunoglobulins from multiple human donors, providing a broad immunological repertoire. The exact mechanisms of IVIG action are not fully understood, but they are known to modulate the expression and function of Fc receptors, interfere with complement activity, disrupt cytokine actions, and inhibit T- and B-cell activation and differentiation (2). IVIGs are indicated for the treatment of autoimmune diseases such as immune thrombocytopenic purpura, chronic inflammatory demyelinating polyneuropathy, multifocal motor neuropathy, and Guillain–Barré syndrome. They are also used in Kawasaki disease and both primary and secondary immunodeficiency. However, a significant downside of IVIG use is its high production cost, which limits accessibility in low-income countries (3). In contrast to polyclonal antibodies, monoclonal antibodies (mAbs) are produced from a cell lineage made by cloning a unique white blood cell. A monoclonal antibody usually binds to a single epitope on the antigen surface. Therapeutic monoclonal antibodies act through multiple mechanisms, such as blocking targeted molecule functions, inducing apoptosis in cells that express the target, or modulating signaling pathways. Many mAbs that act as ISs have been approved for the treatment of different inflammatory diseases. The more widely used mAbs in the clinical practice of treating inflammatory diseases are anti-tumor necrosis factor-alpha (anti-TNF-α), anti-integrin, anti-interleukin IL-12-23, and anti-CD-20 on B lymphocytes (2).

Autoimmunity starts when immune self-tolerance fails (4, 5). This triggers an immune activity against self-antigens, which generally results in collateral damage to the involved tissue(s) and elicits the disease symptoms. Immune response suppression is therefore desired, and both CSs and ISs are prescribed for this purpose in most autoimmune diseases, sometimes as first-line therapies. Their effect may halt further injury to the involved tissue(s), improve symptoms, and induce remission. Examples include systemic lupus erythematosus (SLE) (6), rheumatoid arthritis (RA) (7), and psoriasis (Ps) (8, 9). However, four non-degenerative autoimmune diseases where CSs or ISs are not recommended by treatment guidelines stand out: advanced type 1 diabetes mellitus (T1D) in adults (10), Hashimoto’s thyroiditis (HT) (11), Graves’ disease (GD) (12), and primary biliary cholangitis (PBC) (13, 14). This perspective article discusses the ineffectiveness of CSs and ISs in the treatment of these four autoimmune diseases.

## Autoimmune target tissue destruction and regeneration

**Figure 1:**
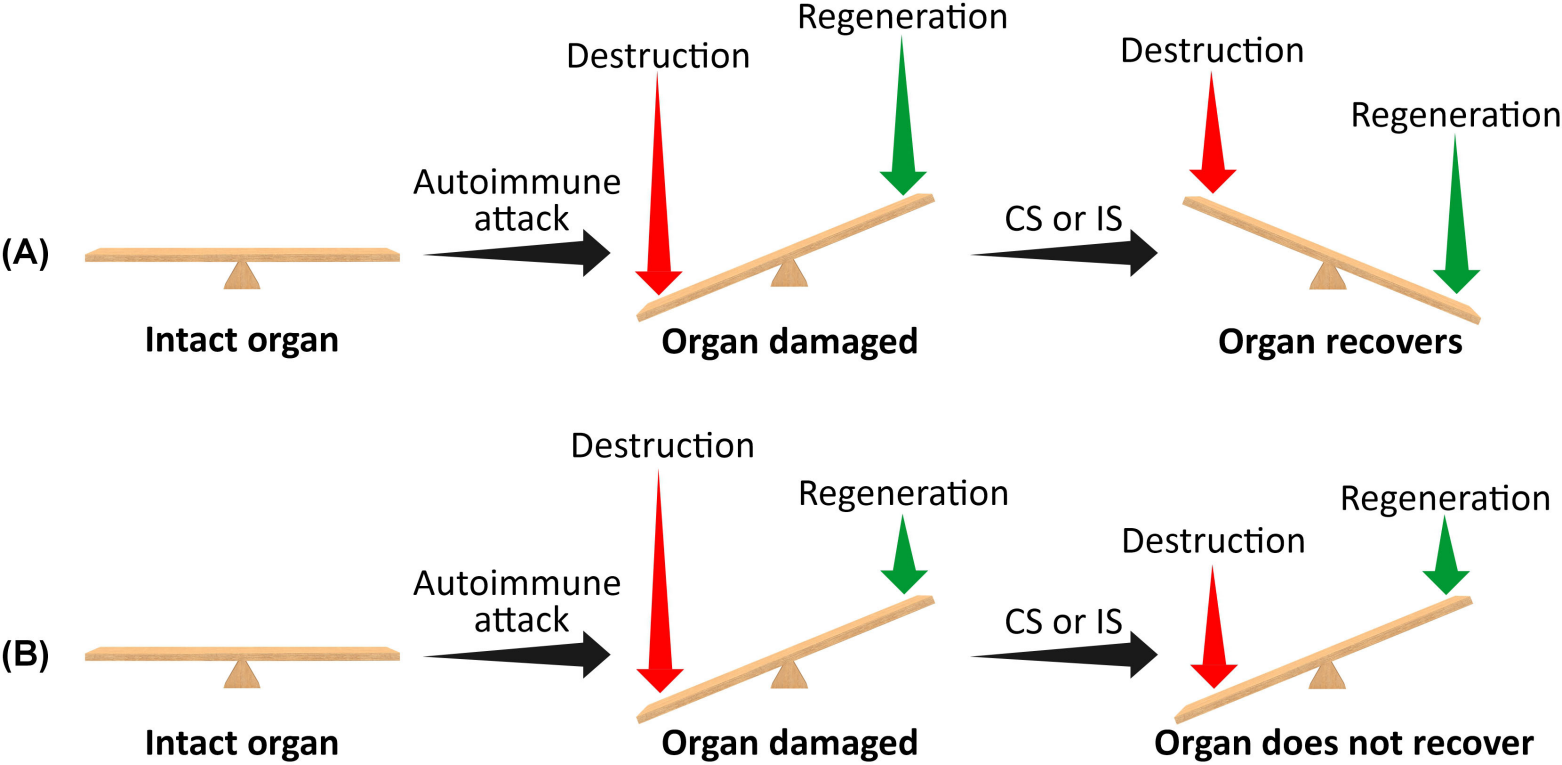
A schematic representation of the effect of corticosteroids or immunosuppressive drugs on organ recovery following an autoimmune attack when the residual mass of target cells is very small (i.e., when the disease is non-degenerative). **(A)** High regeneration rate. **(B)** Very low regeneration rate. The length of the vertical arrows represents the extent of tissue destruction and regeneration rates. When the regeneration rate exceeds the destruction rate, the affected organ will eventually recover, and symptoms will improve following the use of corticosteroids or immunosuppressants. Conversely, if the destruction rate exceeds the regeneration rate, the organ will not recover, and symptoms will persist. CSs, corticosteroids; ISs, immunosuppressants.

When the damage to a tissue targeted by an autoimmune response is profound, reducing or even halting the immune attack with ISs or CSs is ineffective, and symptoms cannot improve until the damaged cells are replaced with new ones. This will occur only when the affected tissue(s) possess the regenerative capacity required for organ recovery. Organ recovery is expected to relieve the severity of the symptoms and induce remission. The following discussion may clarify this point. Define R, the net rate of tissue generation as:


(1) R = Rreg – Rdes

where Rreg is the regeneration rate of the tissue under the autoimmune attack and Rdes is the destruction rate of the tissue due to the autoimmune attack. When R > 0, an autoimmune response should not evoke symptoms since tissue regeneration will exceed tissue loss. Autoimmune symptoms appear when R < 0. This happens when Rreg < Rdes. Under the last condition, all tissue cells will eventually be destroyed if the immune attack continues. Drugs that suppress the immune reaction will cause a decrease in the value of Rdes. Define R1, the net rate of tissue generation under the use of immunosuppressive drugs as:


(2) R1= Rreg – Rdes1

where Rdes1 is the modified destruction rate of the tissue under the use of CSs or IS (Rdes1 < Rdes). Equation 2 assumes that the rate of tissue regeneration (Rreg) is not influenced by the use of CSs or ISs. When R1 > 0, the tissue will recover following CS or IS use, and symptoms will improve over time. However, when R1 ≤ 0, the use of CSs or ISs will not result in tissue recovery and will not alleviate symptoms. Specifically, if the regeneration rate (Rreg) is extremely low, the net rates of tissue generation in the untreated disease or following the use of CSs or ISs will be negative (both R and R1). In this case, the tissue will be more susceptible to damage by the autoimmune assault, and immunosuppressive drugs will not be effective in relieving symptoms (**Figure 1**).

## Both the adult pancreatic islets and the thyroid follicles have negligible regenerative capacities. Therefore, Type 1 diabetes and thyroid autoimmune diseases are refractory to CS or IS treatments

Type 1 diabetes mellitus is the most common chronic autoimmune disease in young patients. It is caused by the destruction of pancreatic endocrine β cells, which are responsible for producing insulin in the islets of Langerhans. As a result, the body becomes deficient in insulin and experiences hyperglycemia. The primary treatment for T1D involves regular insulin injections to control hyperglycemia (15). A defining feature of T1D is the immune system’s recognition of β-cell proteins as autoantigens, leading to an autoimmune response involving autoreactive CD4+ and CD8+ T cells, as well as autoantibodies. Various autoantigens are linked to T1D, including non-specific islet cell autoantigens (ICA), insulin, glutamic acid decarboxylase 65-kDa (GAD65), and islet antigen 2 (IA-2) (16). In young rodents, tissue growth and sprouting occur at the cut surface after a pancreatectomy. Similar tissue growth has also been observed in rare cases involving children post-pancreatectomy. However, the ability for this type of regeneration diminishes significantly in adult animals and is completely absent in adult humans. Animal studies suggest that the exocrine pancreas can regenerate and fully recover from conditions like acute pancreatitis. By contrast, the regenerative capacity of the endocrine islets in adults is limited (17). In particular, the proliferative rate of β cells is quite high in young rodents but declines rapidly with age (18). This raises uncertainty about whether the adult human pancreas can regenerate β cells to a physiologically significant extent (17). Consequently, based on our earlier discussion, ISs are not expected to be effective in treating type 1 diabetes in adults, especially in adults with advanced diseases. As will be discussed later, in the early stages of T1D (pre-clinical stages or newly diagnosed disease), when the β-cell population is not yet fully destroyed, immunosuppressive therapies may help delay disease progression. Corticosteroids, in contrast, induce hyperglycemia and insulin resistance (19), and their use is generally contraindicated in diabetes (20).

Hashimoto’s thyroiditis is a common autoimmune disorder that affects women 7 to 10 times more frequently than men. This condition results from a combination of genetic predisposition, X chromosome inactivation patterns, environmental influences, and microbiome composition, which together disrupt self-tolerance mechanisms. The disorder is characterized by lymphocytic infiltration of the thyroid gland and an antibody-mediated autoimmune response. Specifically, antibodies against thyroid peroxidase antibodies (TPOAbs) play a significant role in the destruction of thyrocytes. Hypothyroidism caused by Hashimoto’s thyroiditis is treated with thyroid hormone replacement agents such as levothyroxine, triiodothyronine, or desiccated thyroid extract (11). The thyroid gland is characterized by its slow cell turnover rate, with cells estimated to divide approximately once every 10 years and approximately five times during adulthood (21, 22). Therefore, CSs or ISs are not recommended for the treatment of Hashimoto’s thyroiditis.

Graves’ disease is an autoimmune disorder that primarily targets the thyroid gland and is the leading cause of hyperthyroidism. It is particularly common among women of reproductive age, though it can occur at any age. The hyperthyroidism in Graves’ disease is driven by autoantibodies to the thyroid-stimulating hormone receptor (TSHR), which act as agonists, prompting excessive thyroid hormone production and disrupting pituitary control of the thyroid gland. These TSHR autoantibodies are also responsible for Graves’ orbitopathy and pretibial myxedema. The treatment options for GD have remained largely unchanged for many years, consisting of antithyroid drugs, radioiodine therapy, or surgery (23). Since GD results in enlargement of the thyroid gland, the rate of tissue destruction, Rdes, is negative, while Rreg = 0, leading to R > 0, as per Equation 1. The rate of thyroid enlargement may be reduced with the use of corticosteroids or immunosuppressive drugs. However, even under the influence of these drugs, the rate of tissue destruction (Rdes1) will remain negative (indicating continued tissue enlargement). The best clinical scenario following treatment is Rdes1 = 0, meaning that there is no further growth of the gland. Whether drug treatment merely slows down thyroid growth (Rdes1 < 0) or stops it completely (Rdes1 = 0), according to Equation 2, R1 ≥ Rreg = 0. Therefore, the gland will not shrink following CS or IS treatments, and their use is not recommended by Graves’ disease guidelines (12).

## The liver possesses an inherently high regenerative capacity. Nevertheless, corticosteroids or immunosuppressants are not effective in primary biliary cholangitis

Primary biliary cholangitis is a chronic autoimmune disorder characterized by cholestasis, primarily affecting middle-aged women. It entails the destruction of intrahepatic bile ducts and the presence of circulating anti-mitochondrial antibodies (AMAs) targeting the E2 subunit of the pyruvate dehydrogenase complex (PDC-E2). The disease progression varies, often lasting for several decades. Ursodeoxycholic acid (UDCA) has demonstrated efficacy as the primary treatment in reducing injury. However, aggressive PBC can lead to complications such as fibrosis, cirrhosis, end-stage liver disease, and death (24). The impairment of the Cl^−^/HCO_3_^−^ exchanger AE2 in cholangiocytes and lymphoid cells leads to biliary epithelial cell (BEC) destruction. This destruction occurs due to reduced biliary HCO_3_^−^ secretion, disrupting the protective alkaline umbrella that typically prevents toxic apolar bile salts from penetrating cholangiocytes (25). The liver is the only solid organ that employs regenerative mechanisms to ensure that it is always at 100% of what is required for body homeostasis (26). During liver regeneration, all hepatic cell types contribute to cell proliferation, without relying on traditional “stem cells”. If hepatocyte or cholangiocyte proliferation is notably impaired, each cell type can transdifferentiate into the other, functioning as a facultative stem cell (26). Considering the earlier discussion and the liver’s high regenerative capacity, it is surprising that treatment options for PBC do not involve CSs or ISs (13, 14). One potential explanation for the ineffectiveness of these drugs in PBC is the impairment of liver regenerative capacity following severe liver disease. Extensive PBC-induced destruction of biliary epithelial cells may eventually result in cholestasis that promotes the destruction of hepatocytes, fibrosis, and cirrhosis (27). Accumulation of bile acid, as typically observed in PBC, induces the expression of proinflammatory cytokines in both mouse and human hepatocytes. This in turn stimulates neutrophil recruitment, leading to hepatocyte necrosis (28). Although the author of the present paper is not aware of any study directly linking PBC to impaired liver regeneration, impaired liver regeneration has been reported in the mouse cirrhosis model. Fibrosis and cirrhosis were induced in mice following acetaminophen (APAP) administration. It seems that APAP liver toxicity in mice is dose-dependent. A low overdose of APAP (300 mg/kg) caused profound liver injury but also triggered significant compensatory regeneration, leading to injury regression and spontaneous recovery. Conversely, a severe overdose (600 mg/kg) notably inhibited liver regeneration, resulting in prolonged injury and reduced survival (29). As per Equation 2, an impaired liver regeneration rate may lead to R1 < 0, potentially explaining the ineffectiveness of CSs or ISs in severe liver disease associated with PBC.

Another mechanism that may explain the ineffectiveness of immunosuppressive drugs in severe PBC is the reduced frequency of CD8+ T cells in late-stage PBC. Cytotoxic CD8+ T cells are the primary effector cells responsible for biliary epithelial cell injury in PBC (30). The number of CD8+ T cells surrounding the damaged bile duct was reported to decrease in late-stage PBC compared to early-stage PBC (30). This observation is supported by the analysis of single-cell RNA (scRNA) sequencing data from CD45+ liver cells of patients with cirrhosis, indicating a significant decrease in CD8+ T cells (and NK cells) compared to healthy controls (31). Therefore, it is possible that late-stage cirrhosis in PBC contributes to the reduced number of cytotoxic T cells. Reduced autoimmune activity in late-stage PBC may result in decreased effectiveness of CSs and ISs in alleviating disease symptoms.

## Discussion

In most cases, autoimmune diseases are diagnosed after the initial onset of symptoms. By this time, the target tissues have already sustained damage, leading to the manifestation of symptoms. If the damage is partial, immunosuppressive drugs can slow or halt the autoimmune attack, prevent further damage, and preserve organ function. Furthermore, if the organ has a high regenerative capacity, slowing down the immune attack with CSs or ISs may shift the balance toward tissue regeneration (Equation 2), replacing injured cells with new ones and potentially inducing remission. However, if the damage is substantial and the regenerative rate is extremely low, halting the immune response offers little benefit. The organ’s function is already compromised, and even complete suppression of the immune reaction will not shift the balance due to the tissue’s low regenerative potential. In such cases, immunosuppressive drugs will be ineffective (**Figure 1**). The clinical symptoms of T1D and HT are observed only after a significant injury to the target tissues has already occurred (32, 33). Extremely low regeneration rate is an inherent property of adult pancreatic islets and the thyroid follicles. Additionally, an extremely low regeneration rate can develop if tissue destruction is significant, such as in late-stage primary biliary cholangitis. In all these conditions, immunosuppressive drugs are not effective.

Another mechanism that may explain the ineffectiveness of immunosuppressive drugs is a low level of autoimmune activity, which may develop following tissue injury. The low number of cytotoxic T cells that infiltrate the liver in advanced PBC may indicate a low autoimmune activity if the cytotoxic activity of these cells is not enhanced at the same time.

As previously mentioned, the refractoriness of T1D in adults with advanced diseases, as well as HT and GD, to corticosteroids or immunosuppressants may be attributed to the significant loss of excreting cells and the low regenerative capacity of the target tissues. The drug resistance of PBC may also be related to severe tissue injury, impaired liver regeneration, or a reduced number of CD8+ T cells, all of which occur only in late-stage PBC. Therefore, similar to early-diagnosed T1D, early-stage PBC may be more responsive to immunosuppressive drug treatment. Several clinical studies investigating the combination of UDCA with CSs and ISs in the treatment of PBC have been published. These studies indicate a moderate to marked improvement in some biochemical markers of liver function in patients treated with the combination therapy, compared to those treated with UDCA alone (34–36). However, none of these studies compared early-stage PBC with late-stage PBC. The author of this work speculates that the biochemical improvements observed in patients treated with the combination therapy were primarily related to a subgroup of patients with early-stage PBC.

An autoimmune degenerative disease (i.e., a disease that increasingly deteriorates over time) may benefit from the use of CSs or ISs even when the regenerative capacity of the affected tissue is negligible. Although symptoms are not expected to improve with immunosuppressive drugs, the progression of the disease may slow down. For example, corticosteroids are commonly used during acute relapses of multiple sclerosis (MS) (37), a degenerative autoimmune disease affecting the central nervous system, a tissue with very limited regenerative potential (38). However, recent findings by Lazzarotto et al. have reported remyelination in MS patients. A review of six studies found that a short course of methylprednisolone for MS patients with acute exacerbation decreased the probability of the condition worsening within the first 5 weeks of treatment (39). Other ISs are also indicated in MS (40). Axonal loss in spinal cord lesions from five paralyzed MS patients averaged 68% (41). Axonal loss in newly diagnosed MS patients is expected to be lower (<68%). In contrast, diseases such as advanced T1D and HT are characterized by a much higher target cell loss. For example, in newly diagnosed T1D patients, pancreatic β-cell loss averaged 90% (32). Advanced T1D is therefore expected to be characterized by an almost complete loss of β cells. This renders the use of immunosuppressants ineffective in protection against further damage in advanced T1D. Regarding HT, the literature describes the loss of thyroid follicular cells as “dramatic” (33), although no supporting data are provided. Nonetheless, the author of this article believes that the destruction of thyroid follicular cells in most HT patients is substantial, and therefore, CSs or ISs may not be effective in halting further deterioration in these patients. Unlike T1D, HT, and GD, the severity of PBC develops over time (42). Therefore, this article recommends the *early* use of CSs or ISs in PBC not only due to the relatively high regenerative potential of the liver at this stage but also because these drugs may slow down the development of advanced diseases.

Guillain–Barré syndrome (GBS) is an acute monophasic polyradiculoneuropathy triggered by an autoimmune attack on the proximal peripheral nerves and nerve roots (43). In contrast, MS typically involves chronic attacks on the central nervous system (CNS). Additionally, GBS is not a degenerative disease; most patients (92%) experience progressive recovery, with spontaneous recovery occurring within 12 months of disease onset (44). While the CNS has a low regenerative capacity, the peripheral nervous system can regenerate following injury. However, unlike in MS, corticosteroids have little impact on GBS (45). This ineffectiveness may be related to the acute nature of the autoimmune assault in GBS.

Collectively, CSs and ISs are effective in chronic *non-degenerative* autoimmune diseases with partial tissue damage and high regenerative potential, such as RA, SLE, and Ps. These drugs are ineffective in chronic non-degenerative autoimmune diseases where damage to target cells is significant and regenerative potential is inherently poor, such as advanced T1D, HT, and GD. However, CSs and ISs are effective in chronic autoimmune *degenerative* diseases with limited damage to target cells, even when the regenerative potential is poor, such as in MS. In *acute* autoimmune diseases, CSs and ISs are ineffective, even when regenerative capacity is high (e.g., GBS). PBC is characterized by the degeneration and necrosis of intrahepatic biliary epithelial cells, which regress over time (42). Therefore, early PBC may be regarded as a degenerative disease, although this term is not commonly used for PBC. Moreover, during early PBC, the liver’s high regenerative capacity is preserved. The author hypothesizes that CSs or ISs are effective in treating this stage. Advanced (end-stage) PBC is not a degenerative condition, as the disease no longer progresses. At this stage, the liver’s regenerative capacity is considerably decreased, and CSs or ISs are not effective. It seems that a degenerative autoimmune disorder is a sufficient condition for the effectiveness of CSs or ISs (as seen in MS or early PBC), while an acute condition will not benefit from these drugs (e.g., GBS). **Table 1** summarizes these data.

**Table 1:** Classification of different autoimmune diseases regarding the effectiveness of corticosteroids or immunosuppressants.

Autoimmune diseases	Chronic disease	Degenerative disease	Regenerative capacity	Target tissue damage	CSs or ISs recommended
RA, SLE, Ps	Yes	No	High	Low	Yes
Advanced T1D, HT, GD, advanced PBC	Yes	No	Low (hypothesized for advanced PBC)	High (not known for GD)	No
Early T1D	Yes	Yes	?	90%	Yes (only ISs)
MS	Yes	Yes	Low	Average < 68%	Yes
GBS	No	No	High	High	No
Early PBC	Yes	Yes	High	Low	Yes (hypothesized)

CSs, corticosteroids; GBS, Guillain–Barré syndrome; GD, Graves’ disease; HT, Hashimoto thyroiditis; ISs, immunosuppressants; MS, multiple sclerosis; PBC, primary biliary cholangitis; Ps, psoriasis; RA, rheumatoid arthritis; SLE, systemic lupus erythematosus; T1D, type 1 diabetes mellitus.

The early stages of T1D (Stages 1 and 2) are marked by the presence of at least two autoantibodies targeting pancreatic β cells, with no dysglycemic states or clinical symptoms apparent yet. At these stages, the pancreatic β-cell population remains largely intact, enabling optimal glycemic control. However, metabolic alterations can be detected through oral and intravenous glucose tolerance tests. During these early stages of T1D, ISs may be able to contain and halt the autoimmune process, extending the duration of stages 1 and 2 and delaying the onset of the clinical phase of diabetes (46).

Teplizumab, an anti-CD3 monoclonal antibody targeting the T-cell receptor (TCR) receptor complex of CD3+ T lymphocytes, was recently approved in the USA to delay the onset of stage 3 T1D in adults and pediatric patients aged 8 and older with stage 2 T1D. Teplizumab’s mechanism of action may involve partial agonistic signaling and deactivation of pancreatic beta-cell autoreactive T lymphocytes (47).

However, not all ISs demonstrate the same efficacy as Teplizumab in delaying the onset of Stage 3 in T1D. Abatacept, a cytotoxic T lymphocyte-associated protein Ig (CTLA4Ig), inhibits T-cell activation by blocking costimulatory signals transmitted through the CD80/86 pathway. The effect of abatacept on the progression of T1D in stage 1 T1D patients was investigated in a randomized, double-masked, controlled trial. Although the C-peptide responses to oral glucose tolerance tests were higher in the abatacept arm, 1 year of monthly abatacept treatment in stage 1 T1D patients did not significantly delay progression to glucose intolerance compared to the placebo group (48).

In autopsy samples from recent-onset T1D patients who died of diabetic ketoacidosis, pancreatic β-cell loss ranged from 70% to 99%, with an average deficit of 90% (32). Therefore, immunosuppressive drug treatment in recent-onset patients (early stage 3) may slow the destruction of the remaining β cells and delay disease progression in those with a higher residual β-cell mass.

Several immunosuppressive monoclonal antibodies have been tested in recent-onset T1D patients.

Otelixizumab, an anti-CD3 antibody, failed to preserve C-peptide levels or other markers of metabolic control in a phase 3 placebo-controlled study involving patients (ages 12 to 45) with newly diagnosed T1D (49).

Rituximab is an anti-CD20 monoclonal antibody that selectively depletes B lymphocytes. Either rituximab or a placebo was administered to 87 newly diagnosed T1D patients (8–40 years old) once a week for the first 4 weeks of the study. After 1 year, the mean C-peptide area under the curve (AUC) was significantly higher in the rituximab group compared to the placebo group. Additionally, the rituximab group showed significantly lower glycated hemoglobin levels and required less insulin (50). However, the beneficial effect of early rituximab treatment began to wane after 2 years (51). Moreover, RNA-sequencing analysis of whole-blood samples from this trial revealed a temporary increase in heterogeneous T-cell populations. This increase was linked to reduced pharmacodynamic activity of rituximab, heightened proliferative responses to islet antigens, and accelerated C-peptide loss, which predicted a faster progression of T1D (52). It can be concluded that early rituximab treatment delays disease progression only in the short term.

Alefacept, a fusion protein composed of two LFA-3 molecules linked to the Fc portion of IgG1, targets CD2, a receptor predominantly found on CD4+ and CD8+ effector memory T cells. In newly diagnosed T1D patients, two 12-week courses of alefacept preserved C-peptide secretion, decreased insulin requirements and hypoglycemic episodes, and resulted in favorable immunologic profiles more than 1 year after treatment ended (53).

Golimumab, a human monoclonal antibody specific to TNF-α, was tested over 52 weeks against a placebo in 84 children and young adults (ages 6 to 21) with newly diagnosed overt T1D. Patients treated with golimumab showed better endogenous insulin production and required less exogenous insulin compared to those who received the placebo (54).

Low-dose anti-thymocyte globulin (ATG), commonly used to treat transplant rejection and consisting of rabbit antibodies against human thymocytes, was assessed in a randomized, double-masked, placebo-controlled phase 2b trial with newly diagnosed T1D patients (ages 12–45). Administering ATG at a dose of 2.5 mg/kg via two intravenous infusions—0.5 mg/kg on the first day and 2 mg/kg on the next—preserved C-peptide levels, reduced glycated hemoglobin (HbA1c), and increased regulatory to conventional T-cell ratios 2 years post-therapy (55).

Overall, some immunosuppressive therapies may effectively delay T1D progression when used in the early stages of the disease (Stages 1 and 2), while the pancreatic β-cell population is still largely intact, or in newly diagnosed patients. For some of these drugs, the benefits of early treatment tend to diminish after a few years.

Autoimmune hepatitis (AIH) is a chronic liver disease characterized by immune-mediated destruction of hepatocytes, leading to inflammation and fibrosis. In contrast to PBC, the standard treatment for AIH involves corticosteroids and azathioprine (56). In severe PBC, both cholangiocytes and hepatocytes are destroyed (27), impairing the regenerative capacity of the liver. In contrast, AIH predominantly affects hepatocytes while sparing cholangiocytes. In cases of chronic liver injury, cholangiocytes can convert into hepatocytes (and vice versa), thereby maintaining the liver’s regenerative capacity (57, 58). This likely occurs in AIH, where hepatocyte regeneration is preserved despite the immune assault on these cells. However, the substantial injury to both hepatocytes and cholangiocytes associated with severe PBC prevents trans-differentiation and hinders liver regeneration. This discrepancy between AIH and PBC may explain the differing effectiveness of CSs and ISs in treating the two conditions. Injury induced in mouse liver ductal organoids by APAP administration led to an upregulated expression of fibrogenic cytokines by cholangiocytes in the organoids, which was associated with increased cholangiocyte apoptosis and decreased cholangiocyte proliferation (59). It can be speculated that the inhibition of liver regeneration induced by high doses of APAP (29) described in the former section was a result of the injury to cholangiocytes and hepatocytes. The extensive injury to both hepatic cell types may be the cause of the impaired liver regenerative capacity reported. Similarly, the severe liver injury model recapitulating some of the key features of clinical acute-on-chronic liver failure (ACLF) resulted in impaired liver regeneration, resembling the decreased liver regeneration observed in ACLF patients. Liver regeneration in this model was severely impaired because of a shift from the activation of the pro-regenerative IL-6/STAT3 pathway to the anti-regenerative IFN-γ/STAT1 pathway (60). IFN-γ signaling upregulation was reported in high-risk PBC patients (patients who do not respond to UDCA) (61). Primary sclerosing cholangitis (PSC), a rare chronic immune-mediated cholestatic liver disease causing inflammation, fibrosis, and destruction of the bile ducts, is also associated with an increased IFN-γ response (62).

Autoimmune hepatitis has two major variant phenotypes in which the features of classical disease are co-mingled with those of PBC or PSC. The frequency of AIH overlap with PBC is 7%–13%, and the frequency of AIH overlap with PSC is 8%–17% (63). Based on the prior discussion, the existence of AIH-PBC or AIH-PSC overlap syndrome is not surprising. When PBC or PSC develops to the extent of highly impaired regenerative potential, hepatocyte tissue becomes more susceptible to irreversible damage following an autoimmune attack, and an overlap syndrome is diagnosed.

Taken together, severe PBC is refractory to immunosuppressive drugs probably due to the destruction of both cholangiocytes and hepatocytes during late-stage diseases. In contrast, the addition of CSs or ISs to UDCA treatment of early-stage PBC may be beneficial.

## Summary

This work attributes the ineffectiveness of immunosuppressive drugs in certain autoimmune disorders to the extensive damage caused by the immune attack, coupled with the limited regenerative capacity of the affected tissues. The low regenerative rate may be an inherent characteristic of the tissue, as seen in adult pancreatic islets and thyroid follicles, or it may be a consequence of the autoimmune assault itself, as in primary biliary cholangitis. A second possible mechanism of refractoriness to immunosuppressive drugs in primary biliary cholangitis involves reduced autoimmune activity in the advanced stages of the disease. It is speculated that adding immunosuppressive drugs to UDCA in the treatment of early-stage primary biliary cholangitis may improve liver function and clinical outcomes. A degenerative autoimmune disorder like multiple sclerosis, which affects the central nervous system, can benefit from corticosteroid treatment despite the CNS’s low regenerative capacity, as a significant number of neurons remain intact in MS. In contrast, a non-degenerative acute autoimmune disease like Guillain–Barré syndrome is typically refractory to corticosteroids, even though the immune attack targets the peripheral nervous system, which can regenerate after injury.

## Data Availability

The original contributions presented in the study are included in the article/supplementary material. Further inquiries can be directed to the corresponding author.
